# Cost-effectiveness analysis of coronary arteries bypass grafting (CABG) and percutaneous coronary intervention (PCI) through drug stent in iran: a comparative study

**DOI:** 10.1186/s12962-023-00426-y

**Published:** 2023-02-15

**Authors:** NourolHoda Fakhrzad, Mohsen Barouni, Reza Goudarzi, Javad Kojuri, Yunes Jahani, Mohammad Tasavon Gholamhoseini

**Affiliations:** 1 Health Services Management Research Center, Institute for Futures Studies in Health, Kerman University of Medical Sciences, Kerman, Iran; 2 Modeling in Health Research Center, Institute for Futures Studies in Health, Kerman University of Medical Sciences, Kerman, Iran; 3 Chairman of Education Development Center, Shiraz University of Medical Sciences, Shiraz, Iran; 4 Department of Biostatistics and Epidemiology, School of Public Health, Kerman University of Medical Sciences, Kerman, Iran

**Keywords:** Costs-effectiveness, Quality-Adjusted Life Years, Markov model, Seattle angina questionnaire, Short form-36 questionnaire

## Abstract

**Background:**

Cost-effectiveness analysis plays a key role in evaluating health systems and services. Coronary artery disease is one of the primary health concerns worldwide. This study sought to compare the cost-effectiveness of Coronary Arteries Bypass Grafting (CABG) and Percutaneous Coronary Intervention (PCI) through drug stent using Quality-Adjusted Life Years (QALY) index.

**Methods:**

This is a cohort study involving all patients undergoing CABG and PCI through drug stent in south of Iran. A total of 410 patients were randomly selected to be included in the study. Data were gathered using SF-36, SAQ and a form for cost data from the patients' perspective. The data were analyzed descriptively and inferentially. Considering the analysis of cost-effectiveness, Markov Model was initially developed using TreeAge Pro 2020. Both deterministic and probabilistic sensitivity analyses were performed.

**Results:**

Compared with the group treated with PCI, the total cost of interventions was higher in the CABG group ($102,103.8 vs $71,401.22) and the cost of lost productivity ($20,228.68 vs $7632.11), while the cost of hospitalization was lower in CABG ($67,567.1 vs $49,660.97). The cost of hotel stay and travel ($6967.82 vs $2520.12) and the cost of medication ($7340.18 vs $11,588.01) was lower in CABG. From the patients' perspective and SAQ instrument, CABG was cost-saving, with a reduction of $16,581 for every increase in effectiveness. Based on patients’ perspective and SF-36 instrument, CABG was cost-saving, with a reduction of $34,543 for every increase in effectiveness.

**Conclusion:**

In the same indications, CABG intervention leads to more resource savings.

## Introduction

Coronary artery disease is one of the primary health concerns worldwide [1]. Coronary heart disease is one of the three leading causes of death in the world [2] and it is predicted that by 2030, 7 out of 10 deaths worldwide will be due to chronic diseases related to cardiovascular disease [3]. Research has shown that one person dies every second due to cardiovascular disorders [4].

Iran is currently experiencing a health transition. Epidemiologically, the burden of cardiovascular disease has changed dramatically over the past three decades. Cancers, accidents and diseases around childbirth, as well as infectious and contagious diseases are the main causes of death in the country. In these circumstances, identifying the burden of diseases caused by major risk factors in the country and reviewing appropriate treatment measures to prevent and treat these diseases is a research priority. In Iran, about one hundred thousand deaths occur annually due to cardiovascular diseases, which accounts for 30% of all deaths [5].

Coronary arteries disease (CAD) is the most common type of heart disease [1] resulting from accumulation of arteriosclerotic plaques in artery walls [2]. This would lead to artery wall thickening, heart failure, angina pectoris, and myocardial infarction [2]. Coronary Arteries Bypass Grafting (CABG) and Percutaneous Coronary Intervention (PCI) are two common treatments for this condition [6]. In spite of clinical similarities, there are some other issues such as cost-effectiveness, and quality of life (QoL) which need to be considered in decision-making. Several related studies have been conducted in developing countries [7, 8]. Although there is a growing trend of these diseases in developing countries, fewer people in modern countries are inflicted [1]; This is partly due to the fact that cardiovascular health care is more limited in low- and middle-income countries [9].

Cost-effectiveness is a type of economic evaluation method in which the degree of health obtained from an intervention is compared with the value of the resources used for it [10]. This analysis summarizes all costs of the program in one number and all its benefits (effectiveness) in a second number; then, based on the relationship between the two numbers, it prescribes rules and regulations for decision-making. Cost-effectiveness analysis can determine the maximum years intended for a specific amount of expenditure following QALY index [11].

Very little is known about the cost and effectiveness of this type of treatment in developing countries despite the widespread outbreak of such diseases therein [8]. Meanwhile, prolonging the life of patients with disability is not appropriate [12]. It seems indispensable that duration of healthy life along with desirability of productive life and welfare should be taken into account by the governments and international communities [12]. Intervention improves both the quantity and quality of life; as such, to compute qualitative and quantitative facets a criterion for combining the facets seems necessary. In view of that Quality-Adjusted Life Year or QALY can be an optimal option. QALY can be interpreted as a criterion resulting from receiver [13]. This is why it is called cost-utility analysis. Personal and societal priorities are of great importance in calculating QALY [14]. In this respect, a number of studies have been conducted [15–21].

Researchers in this study attempted to analyze the cost-effectiveness of two surgical operations, CABG and PCI, through drug stent and QALY index in Shiraz hospitals, Iran.

## Method

This economic evaluation study focused on patient’s perspective. Markov model was used to estimate the cost, and QALY index was employed for each medical strategy. The model was built using TreeAge Pro 2020. Preliminary data were collected from patients within 2 months. After 6 months of intervention, data were collected again from the same patients. The duration of data collection was approximately 2 months before the intervention and 2–3 months after 6 months of the intervention.

### Sampling

All patients who had undergone CABG and PCI through drug stent in south of *Iran (Fars province, Shiraz)*, Iran, in 2014 participated in the study (410 patients). Data collection started in May 2014 and ended in February 2015. The participants were recruited from Shiraz hospitals, where the research also took place (Faqihi hospital, Namazi hospital, & AlZahra hospital), Iran. All patients who referred to the hospital for treatment during May and July 2014 were selected as participants to complete the sample size. These patients were explained about the purpose of the study and consent form was obtained to participate in the study. Four hundred and thirty patients were first chosen in case of any possible reluctance (215 patients for CABG & 215 for PCI). Five and 15 patients refused to participate from CABG and PCI interventions respectively.

In this study, random sampling has been used. The sample size is calculated by Eq. 1.

1 $$n\, = \,\frac{{2\,\left( {Z_{\alpha } \, + \,Z_{\beta } } \right)\,\left[ {sd_{c}^{2} \, + \,\left( {W\,sd_{q} } \right)^{2} \, - \,\left( {2W\,\rho \,sd_{c} \,sd_{q} } \right)} \right]}}{{\left( {WQ\, - \,C} \right)^{2} }}$$

α: The probability of the first type of error is considered to be 0.05 (Z_α_ = 1.64).

β: The probability of error of the second type and β-1 is the test power, which we consider 90% of the test power (Z_β_ = 1.28).

sd_c_: Deviation of the cost criterion in the coronary artery bypass surgery treatment group is 10972 [22].

sd_q_: QALYs standard deviation in coronary artery bypass surgery treatment group is 0.00063 [22].

W: The maximum willingness to pay is $40,000 [22]. Here ρ is the coefficient of correlation between cost and cost. According to experts, the relationship between goods and costs is positive and their value is about 0.3 (ρ = 0.3).

C: Average cost

Q: QALY

WQ-C (or NMB): The same as NMB (Net Monetary Benefit). The lower the NMB, the greater the difference between the two treatments, and the larger the sample size needed to show this small difference. The minimum value of NMB that is important for us and with that value we also say that there is a difference between the two treatments, was considered 3200 based on the opinion of experts [22].

In this study, costs and QALY indices were calculated regarding a three-percent discounting rate [8]. It took nearly 6 months and the patients' statuses were studied in three consecutive years of 2012, 2013, and 2014. It took 6 months to compare the cost and utility of these two medical procedures.

A follow-up study was then conducted to assess direct/indirect medical cost and health-related quality of life (HRQOL) issue in patients undergoing CABG/PCI using stents. They became aware of the purpose and method of the study through some oral and documented information and they all consented to taking part in the study. Patients filled the questionnaires themselves. Note that in the case of patient failure, a trained interviewer filled the questionnaire in a face-to-face session.

### Decision analysis model

**Fig. 1:**
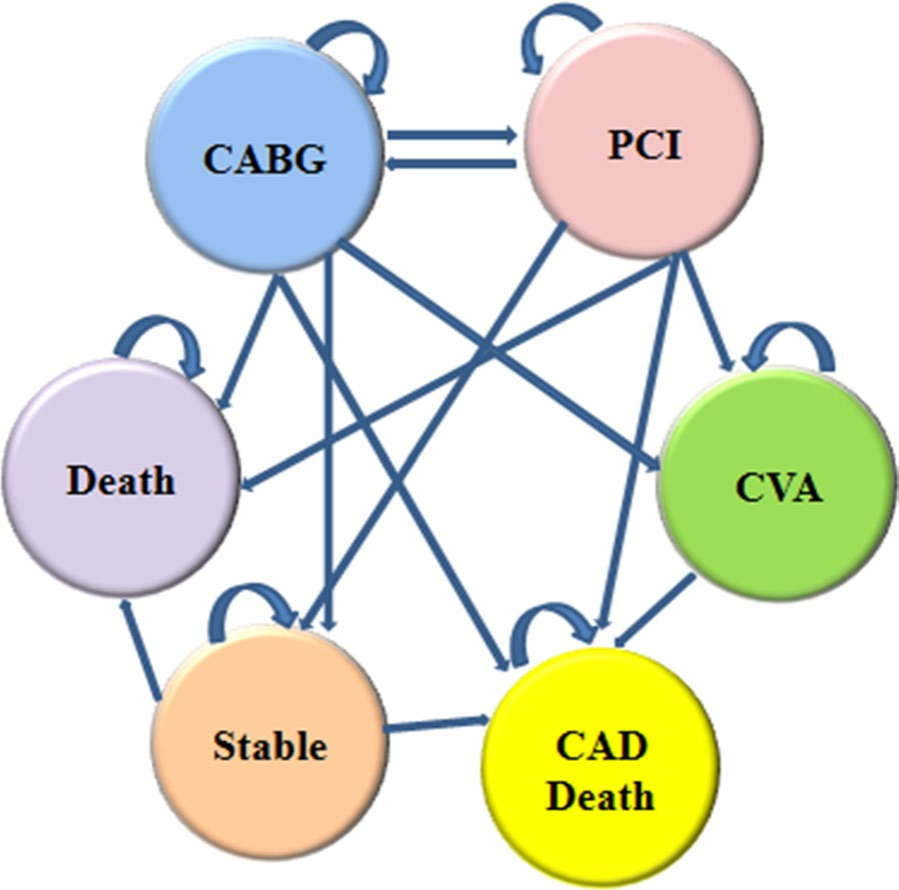
Markov model used for coronary diseases

Markov model was developed based on clinical and economic specialists’ views to be used in the study (Fig. 1). In effect, there are six state following CABG/PCI treatments; CAD death, death, cerebrovascular accident (CVA), and stay on PCI or CABG. Also the model used a 6-month cycle length.

The hazard ratio of repeat revascularization by CABG/PCI after 6 months was extracted from previous studies and used in the model. The transition probabilities of the revascularization for each cycle was calculated using p = 1-e^−rt^: where, p stands for probability, e for natural algorithm, r for occurrence rate, and t for time [8]. The mean age of patients with coronary artery disease was 59 years, so we assumed the patients entered the model at 59 years of age. Accordingly, the initial age was selected based on the mean age of the patients.

### Effectiveness

Data related to utility were collected using the short form of 36 items (SF-36) and Seattle Angina questionnaires (SAQ) (Table 1). The Persian version of SF-36 is used to evaluate health-related Quality of Life (QoL) with 36 items in eight health dimensions, including physical function, role limitations due to physical health, bodily pain, general health, energy, social function, role limitations due to emotional problems, and emotional well-being. Each scale contains degrees from zero to 100, where zero is the minimum and 100 is the maximum status of the intended variable [20].

**Table 1:** The basic cases used in the cost-effectiveness analysis model of CABG and PCI

Variable		Value	Range	References
Probability				
Probability of CABG after CABG		0.006	(0.0054–0.0066)	Calculated
Probability of CABG after PCI		0.1505	(0.13545–0.16555)	Calculated
Probability of CAD Death after CABG		0.0371	(0.03339–0.04081)	Calculated
Probability of CAD Death after PCI		0.0035	(0.00315–0.00385	Calculated
Probability of CAV after CABG		0.001	(0.0009–0.0011)	Calculated
Probability of CAV after PCI		0.0071	(0.00639–0.00781)	Calculated
Probability of death after CABG		0.003	(0.0027–0.0033)	Calculated
Probability of death after PCI		0.0035	(0.00315–0.00385)	Calculated
Probability of PCI after CABG		0.005	(0.0045–0.0055)	Calculated
Probability of PCI after PCI		0.0537	(0.04833–0.05907)	Calculated
Probability of Stability after CABG		0.9476	(0.85284–1.04236)	Calculated
Probability of Stability after PCI		0.7813	(0.70317–0.85943)	Calculated
Probability of CADdeath after stable		0.009	(0.008–0.012)	[23]
Probability of CADdeath after CVA		0.074	(0.059–0.089)	[24]
Probability of death from other causes		0.004	(0.004–0.006)	[25]
Effectiveness				
SAQ	CABG after PCI	−0.357	(−0.3928–(−0.3214))	Calculated
	PCI after PCI	0.079	(0.063–0.094)	Calculated
	CVA after PCI	0.390	(0.312–0.468)	Calculated
	Stable after PCI	0.063	(0.050–0.075)	Calculated
	CABG after CABG	0.158	(0.126–0.189)	Calculated
	PCI after CABG	0.105	(0.084–0.126)	Calculated
	CVA after CABG	0.390	(0.312–0.468)	Calculated
	Stable after CABG	0.135	(0.108–0.163)	Calculated
SF-36	CABG after PCI	−0.008	(−0.009–(−0.006))	Calculated
	PCI after PCI	0.107	(0.085–0.128)	Calculated
	CVA after PCI	0.390	(0.312–0.468)	Calculated
	Stable after PCI	0.063	(0.050–0.076)	Calculated
	CABG after CABG	0.154	(0.123–0.185)	Calculated
	PCI after CABG	0.139	(0.111–0.167)	Calculated
	CVA after CABG	0.390	(0.312–0.468)	Calculated
	Stable after CABG	0.100	(0.080–0.120)	Calculated
Cost(USD)				
CABG after CABG		1880	(1504–2256)	Calculated
PCI after CABG		1128	(903–1354)	Calculated
CVA after CABG		323	(259–388)	Calculated
CADdeath after CABG		3931	(3144–4717)	Calculated
Stable after CABG		2426	(1941–2912)	Calculated
Death after CABG		5700	(4560–6841)	Calculated
CABG after PCI		2086	(1669–2503)	Calculated
PCI after PCI		3365	(2692–4038)	Calculated
CVA after CABG		723	(578–867)	Calculated
CADdeath after PCI		4219	(3375–5062)	Calculated
Stable after PCI		3193	(2555–3832)	Calculated
Death after PCI		4219	(3375–5062)	Calculated

SAQ is a special instrument to assess quality of life in cardiovascular patients. It contains 19 items in five different aspects including physical limitation, angina stability, angina frequency, treatment satisfaction, and disease perception. The scale ranges from zero to 100 where zero is the worst and 100 is the best medical condition [20].

The patients' consent was obtained through questionnaires prior to the medical intervention. They were informed that they would be invited to answer the same questions again in future. After 6 months of medical intervention, the patients were debriefed via these questions again on a phone call and their responses were reanalyzed.

Considering the different socio-economic situation around the world and its effect on the utility, we calculated the utility to make the results of our study real and did not use the findings of previous studies. According to a study conducted in Iran, the socio-economic status affects the utility [26].

To determine the degree of effectiveness based on QALY index, the scores from each questionnaire were processed using QALY formula. A QALY is the years of life adjusted for quality [27]. It is multiplied by the number of the years added based on an objective and standard coefficient ranging from 0 to 1. This is the quality of life (QoL) in relation to health in a specified period. It is presented as follows (Eq. 2):

2 $$QALY sgained = Q^{i} \frac{{1 - e^{{ - rL^{i} }} }}{r} - Q\frac{{1 - e^{ - rL} }}{r}$$

Q^i^ is the lifetime along with post intervention quality, L^i^ denotes the period in which one's life is affected by the treatment, r represents the discounting rate, and e shows Napier's constant. Q and L are already mentioned factors prior to the medical intervention [27].

### Cost

The total cost was analyzed based on the patients’ perspective. It consisted of all direct medical cost (hospitalization & medication) and tariffs. The amount the patient was required to pay was calculated using the hospital bills (Table 1). A discounting rate (3%) was used to show their current monetary value [28].

To estimate the expenses, the patients' medical profiles were used. The cost of medical services, medication, laboratory, diagnostic tests, hospitalizations, and all other treatments they received were calculated.

To make it comparable on an international scale, all costs were calculated in USD. Note that, based on the reported exchange rate from Iran Central Bank, a dollar was rated 26509 Rials in 2014 [29].

All direct/indirect costs (based on the patients’ perspective) for each medical intervention are reported in Table 3. This included all direct medical costs such as hospitalizations and medicines the patient will use, along with direct non-medical cost including travel cost and hotel stay cost, plus indirect costs such as lost productivity for patient and family. The patient’s share was calculated using the sum of the costs incurred from the patients’ hospital bill. The cost of the medicine was calculated by first calculating the cost of the drug for each patient for 6 months followed by the total cost for each treatment. The total cost was then multiplied by 2 to estimate the annual cost of the drug and then discounted at a 3% discount rate for 10 years. Lost productivity for patient and family was also calculated based on the minimum wage for 2014. The minimum wage in 2014 was US$269 and the maximum wage in 2014 was equal to US$353; the average of these two numbers is equal to US$311. The cost of absenteeism for each patient was obtained through multiplying the number of hospitalization days by US$311 and then dividing by 26 working days per month. Finally, the cost of absenteeism was calculated via adding the total cost of absenteeism to patients. The incremental cost effectiveness ratio (ICER) is the outcome of cost-effectiveness approach. When considering whether to fund a new procedure, the ICER can be used to guide decision-making. The cost-effectiveness of each medical intervention is calculated using Eq. 3,^1^.

3 $$ICER=\frac{\Delta Cost}{\Delta Effect}$$

The ACER shows the total costs of an intervention per health outcome achieved, as compared with a baseline situation, which in many cases would be the current situation (Eq. 4) 2.

4 $$ACER=\frac{Cost}{Effect}$$

Following Eichler et al. study, three times Gross Domestic Product (GDP) per capita can be used as a threshold to determine the cost-effectiveness [30]. If the ICER is less than the three times GDP per capita, that intervention is assumed to cost- effective. The GDP per capita in 2012 was 8,329 USD based on the World Bank [31].

### Sensitivity analysis

For sensitivity analysis, initially the tornado diagram was drawn according to which sensitivity analysis was performed for the parameters with the greatest impact on the cost-effectiveness analysis. Next, a one-way sensitivity analysis was performed on parameters.

Finally, a probabilistic sensitivity analysis (PSA) was carried out to determine the effect of all parameter uncertainties simultaneously within the model using Monte Carlo simulation, with a generation of 5,000 trials. Ranges for variables were based on actual data and literature values (Table 1). Cost and health utilities varied based on their actual 95%confidence intervals. We assigned Gamma distribution for cost and beta distribution for transition probabilities plus health utilities.

## Results

Four hundred and ten patients participated in this study. Initially, 430 patients were chosen in case of any possible reluctance (215 patients for CABG & 215 for PCI). Five and fifteen people refused from CABG and PCI studies respectively. Baseline characteristics of the studied patients are summarized in Table 2. The two groups were tested for significant differences regarding sociodemographic and main comorbidity as well as clinical variables. The groups were considered equivalent with no significant difference [32].

**Table 2:** Baseline clinical characteristics

Variable		Coronary arteries bypass grafting (CABG) (n = 210)	Percutaneous coronary intervention (PCI) (n = 200)	P-value
Age mean ± SD		60.23 ± 12.53	58.65 ± 11.19	
Sex (%)	Female	73(36.5)	74(37.4)	0.857
	male	127(63.5)	124 (62.6)	
Marital Status (%)	Married	128 (64)	174 (87.9)	< 0.001
	Single	21 (10.5)	14 (7.1)	
	Widowed	51 (25.5)	10 (5.1)	
Insurance (%)	Medical service organization	115 (57.5)	124 (62.6)	0.559
	Social welfare organization	78 (39)	67 (33.8)	
	Other	7 (3.5)	7 (3.5)	
Diabetes mellitus (%)		79(39.5)	30(15.2)	< 0.001
History of hypertension (%)		69(34.5)	50(25.3)	0.044

Direct medical cost and direct nonmedical cost as well as indirect cost for each intervention presented in Tables 3. As shown, the patients have to pay more expenses for Travel/Hotel stay, and Lost productivity for patient and family in CABG, while medication cost is far higher in PCI. Generally, the patients pay far more expenses in CABG.

**Table 3:** Costs of the CABG&PCI (USD $)

Cost catagory	Cost title	CABG		PCI	
		Total cost	Per capita cost	Total cost	Per capita cost
Direct medical costs	Hospitalizations	67567.1	321.74	49660.97	248.3
	Medications	7340.18	34.95	11588.01	57.94
Direct nonmedical costs	Travel/Hotel stay	6967.82	33.18	2520.12	12.6
Indirect costs	Lost productivity for patient & family	20228.68	96.32	7632.11	38.16
Total		102103.8	486.2	71401.22	357

The average hospitalization time in CABG equals 8.05 ± 5.438 days and 3.19 ± 2.843 in PCI. The total cost of 1 day hospitalization is 67567.1 USD in CABG and 49660.97 in PCI showing the fact that the patients incur more expenses in CABG on average. Per capita cost equals 486.2 USD in CABG and 357 in PCI.

Two general and specific instruments for evaluating quality were employed to analyze the effectineness. Cost-effectiveness ratio was analyzed for each intervention using Eq. 2. The results from Table 4 reveals that PCI is costly and less effective compared to CABG.

**Table 4:** Cost-effectiveness analysis for the base case

	Strategies	QALYs	Incremental QALYs	Costs($)	Incremental Costs	ICER		ACER
SAQ	PCI	1.57	–	85634	–	–		54544
	CABG	3.33	1.75	56618	−29016	−16581	Dominant	17002
SF-36	PCI	1.68		85634		–		50973
	CABG	2.52	0.84	56618	−29016	−34543	Dominant	22467

From the patients’ perspective and SAQ instrument, CABG was cost-saving, with a reduction of $16,581 for every increase in effectiveness. Table 4 reveals that CABG is more effective than PCI. Based on patients' perspective and SF-36 instrument, CABG was cost-saving, with a reduction of $34,543 for every increase in effectiveness.

### sentivity analysis

**Fig. 2:**
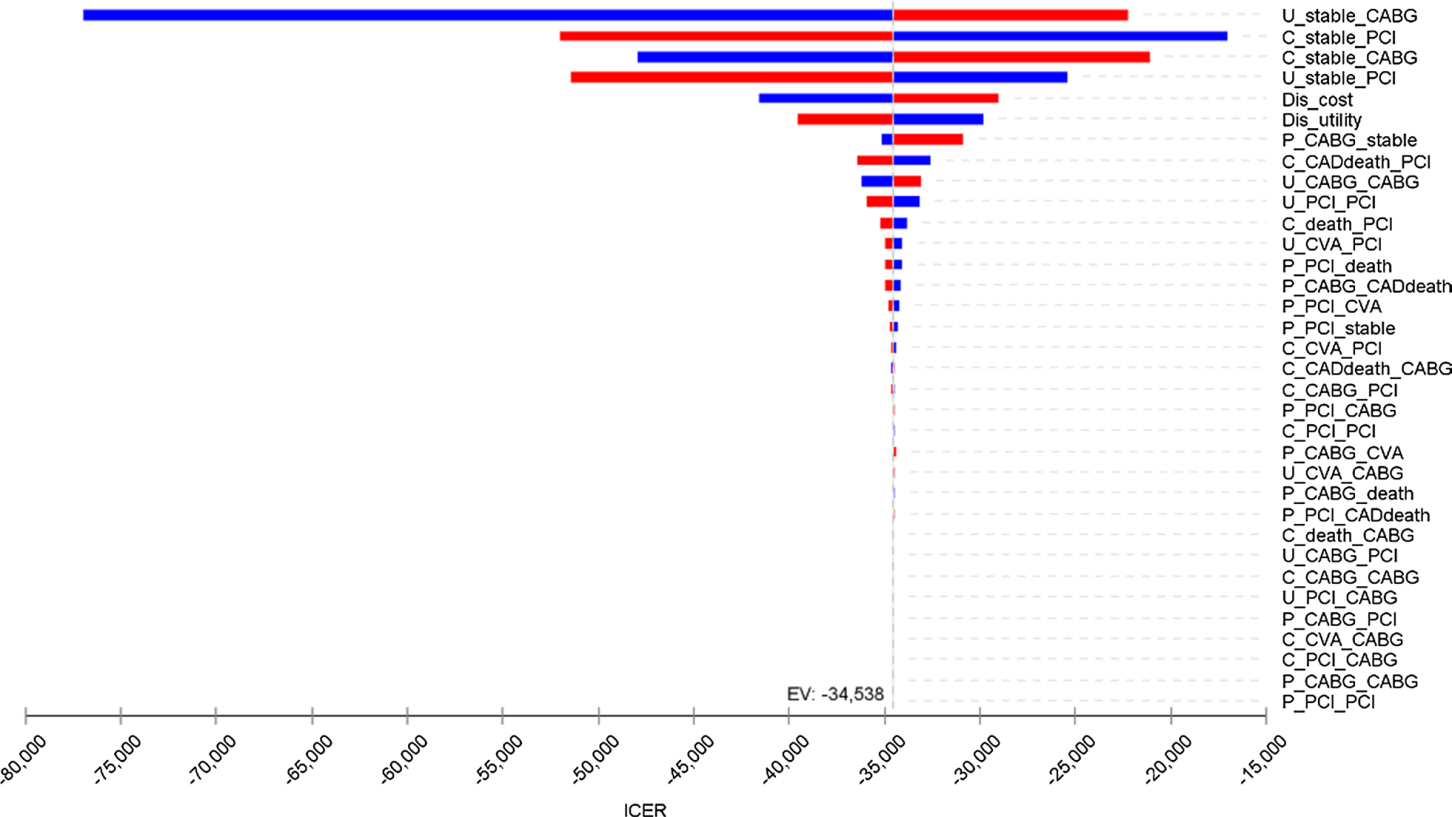
Tornado plot for sensitivity analysis

**Fig. 3:**
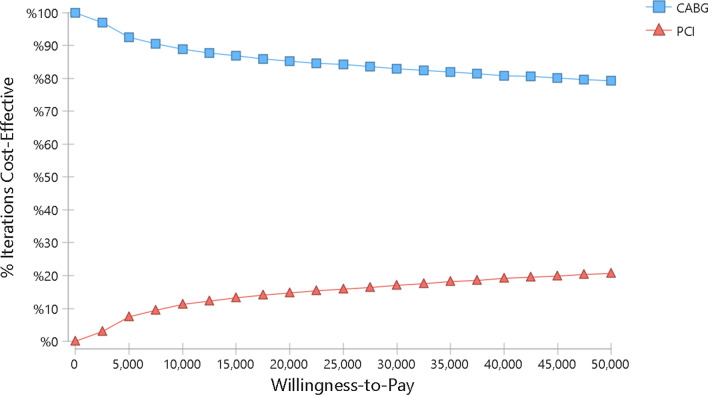
Cost-effectiveness acceptability curve (QALY)

The result of one-way sensitivity analysis is presented as a tornado plot (Fig. 2). Deterministic sensitivity analysis (DSA) findings using the Tornado plot indicated that changing any of the parameters had no effect on the cost-effectiveness outcome. The results of the cost-effectiveness acceptability curve are presented in Fig. 3.

**Fig. 4:**
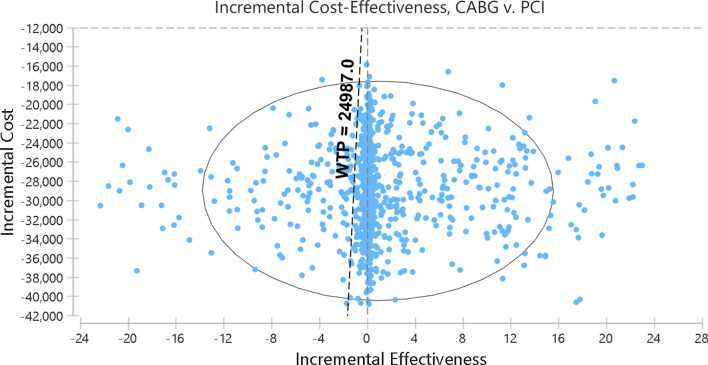
Probabilistic sensitivity analysis, average annual cost, and quality-adjusted life year (QALY)

The results of the probabilistic sensitivity analysis are outlined in Fig. 4. Incremental cost-effectiveness scatter plot showed that most (more than %85) of the trial points were located in the acceptance area (Fig. 4).

## Discussion

This study sought to compare the cost and effectiveness of coronary arteries bypass grafting (CABG) and Percutaneous coronary intervention (PCI) in south of *Iran (Fars province, Shiraz)* in 2014. Markov model as well as QALY index were used to analyze the outcomes of each intervention. To make the findings comparable to previous or future studies, all expenses were calculated in dollar currency (viz. USSD). Following Iran central bank reports, the exchange rate for one dollar was 26,509 Rials and 33,813 Euro on average [28]. The results indicated that CABG enjoys more effectiveness and costs compared to PCI.

The findings reported by Javanbakt revealed that CABG costs 5187 dollars and PCI 4833 dollars. The discounted efficacy of CABG would reach 3.8 in a 5-year period, 6.4 in 10 years, and 8.74 for the entire lifetime based on QALY index. Concerning PCI, this is 3.88, 6.33, and 8.33 for 5 years, 10 years, and the entire lifetime period respectively. The cost- effectiveness ratio of both interventions would reach 28,099 USD $ in a 5-year period. PCI intervention using stents is a far better idea in 10 years and the entire lifetime. All computations have been based on the data collected in 2011 [8]. According to the findings of this study, CABG enjoys more effectiveness and cost, in accordance with the results from Javanbakt.

In another study conducted by Yuck, bypass surgery was cost-effective and more efficient compared to other coaxial treatments including stents (the discounting rate was three percent based on American dollars in 2000). The total cost amounted to 135,200 USD $ for CABG and 135,500 for PCI. The assumed efficacy level was 9.77 for CABG and 9.42 for PCI [15]. As seen, cost-effectiveness ratio is lower in CABG compared to PCI. Our findings contradict those of Yuck since he did it over a longer period. This would necessitate re-use of stents for PCI-treated patients which in turn would yield more cost and less effectiveness.

Serious et al. (2003) showed that the total cost of CABG was 10,653 USD $and PCI 6441 USD $. CABG efficacy was 68 ± 20 (in a 0–100 range) and PCI (in the same range) was 69 ± 20 based on QALY index. The findings of this study showed the outcomes after 1 year. PCI was more cost-effective compared to CABG. They were almost the same regarding death, stroke, and myocardial infarction [19]. These do not concur with our results.

In another study done by Cohen et al. CABG cost 39,241 USD $ yearly, and PCI 30,797. This would amount to 53,260 and 47,641 USD $ for a 5-year period. Usefulness of CABG and PCI would amount to 0.789 and 0.813 for 1 year respectively reaching 3.914 and 3.870 for a 5-year period [15]. As seen, PCI is more affordable for both 1 and 5 year periods while also being more effective in the same length. Our findings are not in line with with Cohen’s study.

Based on Krenn’s study, the total value for CABG patients was higher after 5 years. The results revealed that CABG was more efficient but more costly in patients suffering from multiple coronary arteries disorders [16]. The findings of this study are in line with these results.

Vieira’s study indicated that the medical treatment for both event free and non-event free survival angina cost 3.79, 2.07 for PCI, 2.77, 3.59 for CABG, and 2.81, 4.4 (Based on QALY index) in a 5-year period. Event-free survival cost 9071 dollars for medical treatment, 19,967 USD $for PCI, and 18,263 USD $for CABG. Accordingly, CABG is more cost-effective compared to medical treatment or PCI [7], signifying the convergence of the result of this study with theirs.

Based on Magnuson's study, the total cost for a full lifetime analysis in CABG reached 118,664 USD $ and 109,179 USD $ for PCI. Effectiveness of CABG was 10.355 and PCI 10.004 based on QALY index. This study took 5 years starting in 2005. Both cost and effectiveness were calculated using a 3% discounting rate for each year. According to these findings, CABG is more affordable compared to PCI using stent, in spite of its higher initial cost [17]. These are also similar to our findings confirming the fact that CABG is more effective but more costly compared to PCI. CABG sounds more costly in its initial stages just like what was observed here. Note that Magnuson conducted the study in a far longer period, therefore, there was a need to perform PCI again which made it more costly. PCI incurred more cost and was less effective in their study; therefore, the findings of this study are in line with these results.

The sensitivity analysis revealed that the change of possibilities, cost, and effectiveness had no impact on the results.

This article had some limitation. It was not possible to calculate the marginal cost including settling and transfer; so, these were calculated using questionnaires and oral interviews. Despite the fact that the literature and experts do not recommend a time period of less than 1 year for evaluating interventions, the experts of this article suggested a time period of 6 months, which can be considered as a limitation of our work.

This study had several great advantages over previous studies as a unique model was designed here for the purpose of economic evaluation in cardiovascular patients. Two technical quality instruments, namely SAQ and SF-36, were used to evaluate the effectiveness. Thus, it was possible to guarantee the reliability of their use for such patients. TreeAge Pro 2020 which is used for economic modeling was also employed. It can analyze each probability status in the model for Iranian society which was not regarded in previous studies. At the end of the article, we suggested that a study should be done that conducts a sensitivity analysis on diabetics and elderly patients separately, that the effect measure the interventions on these groups. It is true that CABG is the dominant strategy, but it is better to conduct a linear regression to examine the influencing variables in future studies.

The results indicated that CABG is more cost-effective compared to PCI. In the same indications, CABG intervention leads to more resource savings. The sensitivity analysis revealed that changing probabilities, costs, and effectiveness cannot affect the obtained results.

## Data Availability

All data analyzed during this study may be obtained from the corresponding author [in Persian].

## Footnotes

1 Incremental cost effectiveness ratio.

2 Average cost effectiveness ratio.
